# Combined intervention of swimming plus metformin ameliorates the insulin resistance and impaired lipid metabolism in murine gestational diabetes mellitus

**DOI:** 10.1371/journal.pone.0195609

**Published:** 2018-04-20

**Authors:** Liping Huang, Pingping Yue, Xuefei Wu, Ting Yu, Yang Wang, Ji Zhou, Derun Kong, Keyang Chen

**Affiliations:** 1 Department of Maternal, Child & Adolescent Health, School of Public Health, Anhui Medical University, Hefei, China; 2 Department of Gastroenterology, First Affiliated Hospital, Anhui Medical University, Hefei, China; 3 Department of Hygiene Inspection and Quarantine, School of Public Health, Anhui Medical University, Hefei, China; Max Delbruck Centrum fur Molekulare Medizin Berlin Buch, GERMANY

## Abstract

Gestational diabetes mellitus (GDM) has short- and long- term influence on pregnant women and fetus. Swimming, as an aerobic exercise, can effectively improve the blood glucose level in GDM, but the effect of mild swimming alone was not very substantial. Metformin, as an oral antidiabetic drug, has obvious hypoglycemic effect, and is economic also, but the long-term effect on pregnant women and fetus has not been completely clear. We hypothesize that combined intervention of mild swimming and low dose of metformin, may effectively reduce blood glucose, improve the pregnancy outcomes in GDM dams, but simultaneously avoiding the adverse effects caused by overdose of drug and impotence of mild swimming. The streptozotocin was used to stimulate C57BL/6J mice to develop GDM, by which serum glucose, TC, TG, LDL-C were increased significantly, meanwhile HDL-C was decreased significantly in the GDM control (DC) group compared with the normal control group. Swimming or metformin intervention slightly or moderately improves hyperglycemia, insulin sensitivity and lipid metabolism both in liver and skeletal muscle from GDM mice, while combined therapy of swimming plus metformin markedly ameliorated hyperglycemia (FPG, decreased by 22.2–59.5% from G10 to G18 versus DC group), insulin sensitivity (2.1 and 2.8 fold increase, respectively, in AKT activity versus DC group) and *de novo* lipogenesis (3.2 and 7.0 fold decrease, respectively, in ACC activity, and 1.94 and 5.1 fold decrease, respectively, in SREBP2 level, versus DC group) both in liver and skeletal muscle from GDM mice. We conclude that the combined intervention by metformin plus swimming may be more effective than single action to ameliorate glucose and lipid metabolism *via* improving insulin sensitivity in GDM mice.

## Introduction

In recent years, the morbidity of gestational diabetes mellitus (GDM) is increasing [1]. Approximately 1–18% of all pregnancies are complicated by GDM, depending on the population studied and the diagnostic tests employed [2–3]. It has been defined as any degree of glucose intolerance with onset or first recognition during pregnancy, and associated with insulin resistance (IR) and a great possibility of increased fetal-maternal morbidity as well as short and long-term adverse consequences for the mother and offspring [4–8]. Specifically, the children of women with GDM are more likely to develop overweight or obesity, impaired glucose tolerance and type II diabetes in later life [9–11]. In addition, the presence of GDM always accompanies an increased maternal risk for preeclampsia, cesarean section, and with an increased risk of impaired glucose tolerance and type II diabetes in the years following pregnancy [12].

Traditionally, diet and/or drug are the therapeutic resource most used to control blood glucose concentrations during pregnancy. However, management with diet or drug therapy alone does not fully rectify peripheral insulin resistance for GDM patients. Physical activity has long been known to affect overall metabolism, especially for blood glucose and lipid profiles by direct or indirect effects on insulin action, which involves muscle glucose uptake and glycogen synthesis [13–15]. The American College of Obstetricians and Gynecologists recommends 20–30 minutes of moderate exercise per day on most or all days of the weeks during pregnancy and advises that exercise during pregnancy can lower glucose levels in women with GDM [16]. Exercise is a kind of healthy behavior with good potential to treat or prevent GDM [17]. Recently, some epidemiology studies have reported that physical activity before pregnancy or during pregnancy is associated with a reduced risk of GDM [18–20]. It has been suggested that swimming be one of the most suitable exercises for women during the gestational period [21]. Compared with the physical activity on land, the body in swimming pool has a physiological adaptation to water and puts less weight on bones and ankles than other sports [22]. In addition, swimming helps regulate body temperature and minimizes alterations of the uteroplacental blood flow and core temperature that are induced by exhaustive exercise [23–24]. For these reasons, we chose a swimming program for the pregnant mice.

If maternal normoglycemia cannot be achieved by exercise and lifestyle changes, medication will be needed. Metformin, an oral biguanide reduces hepatic glucose production and enhances peripheral insulin sensitivity, with both direct and indirect effects on liver and muscle [25]. Meanwhile, several epidemiological studies indicated that metformin is an effective and safe alternative treatment for women with GDM [26–28]. But a few studies have found that compared with insulin, pregnant women who have received metformin have a slightly lower gestational age at delivery and more preterm birth [26, 29]. Nevertheless, there still has no study so far examining the actions of metformin in combination with swimming on GDM. Swimming, as an aerobic exercise, can effectively improve the blood glucose level in GDM, but the effect of mild swimming alone was not very substantial. Metformin, as an oral antidiabetic drug, has obvious hypoglycemic effect, and is economic also, but the long-term effect on pregnant women and fetus has not been completely clear. We hypothesize that combined intervention of mild swimming and low dose of metformin, may effectively reduce blood glucose, improve the pregnancy outcomes in GDM dams, but simultaneously avoiding the adverse effects caused by overdose of drug and impotence of mild swimming. In our current study, we found that metformin plus swimming may be more effective than single action to ameliorate glucose and lipid metabolism *via* improving insulin sensitivity in GDM mice.

## Materials and methods

### GDM mouse model

Animal procedures were approved by the Animal Care and Use Committee, faculty of science, Anhui Medical University, in accordance with the international guiding principles for biomedical research involving animals of CIOMS.

The C57BL/6J mice (8 weeks old; male mice: 22–24g; female mice: 17–19g) were purchased from Beijing Vital River, whose foundation colonies were all introduced from Charles River Laboratories, Inc. All mice were maintained under specific-pathogen-free (SPF) conditions, and housed in controlled environment with temperature of (23±2)°C and relative humidity of (50±5) % with a 12h light/dark cycle. Food (Beijing Huafukang Bioscience Co, LTD, Beijing 11027182) and water were available ad libitum before starting experiments. After a week of adaptation, female mice were placed together with males at a ratio of 2:1. Mating was confirmed by the presence of a vaginal mucous plug in next morning, defined as gestational day (GD) 0. The total of successful mating was 73 over the same period, among which 63 dams were administered with streptozotocin to create GDM models, while the remaining 10 dams served as normal controls (NC). Specifically, the GDM mouse model was established by intraperitoneal injection of streptozotocin as 40 mg/kg/per mouse [30] on GD6, GD7 and GD8, respectively, to ensure getting successful. The diabetic state was confirmed when the fasting blood glucose concentration exceeded 11.1 mmol/l (200 mg/dl) and the normal control group mice received 0.9% saline. The glucose levels in mice were detected at GD0, GD2, GD4, GD6, GD8, GD10, GD12, GD14, GD16, GD18. The successful models were 40 of mice, those were randomly distributed into 4 groups (10 animals/group): GDM control (DC), GDM treated with swimming (DS), GDM treated with metformin (DM) and GDM treated with swimming plus metformin (DSM).

Dams were euthanized on GD18 by intraperitoneal injection of ketamine (100 mg/k g), acepromazine (10 mg/kg) and xylazine (100 mg/kg), placentas, liver tissues and skeletal muscle tissues were immediately collected.

### Swimming intervention

The swimming mice were exposed previously to water at a depth of 10 cm at 32±2°C for 10 min to avoid the stress, then add warm water to the depth of 40 cm. Besides, the groups without swimming were not exposed to water as did in exercised groups. Swimming was executed 20 min/day [22] from GD11 to GD18 in a plastic tank with 55 cm in height and 150 cm in diameter. Warm water was added if the water temperature dropped below 30°C. Mice were continuously monitored during swimming to prevent them from drowning. At the end of each daily swimming session, each mouse was dried with a towel until their fur was completely dried before mice being returned to their cages.

### Metformin therapy

The mice in DM and DSM groups were intervened by oral gavage of enteric metformin-hydrochloride tablets as 200 mg/kg per mouse from GD11 to GD18.

### Combined intervention

The mice in DSM group were treated with combined intervention of swimming plus metformin from GD11 to GD18. The dose used and treatment pattern were exactly the same as those aforementioned.

### Maternal blood and tissue sample collection

On GD18, maternal blood samples were collected from the orbital venous plexus after 12h fasting and mice were anesthetized with ketamine (100 mg/kg), acepromazine (10 mg/kg) and xylazine(100 mg/kg). Whole blood was allowed to clot and then centrifuged at 3,000 rpm at 4°C for 20 min to obtain serum, which was kept at -80°C. One third of livers per mice were sliced and preserved in RNAlater for gene expression studies. The remaining livers were immediately removed and rinsed with cold phosphate-buffered saline (PBS) and stored at -80°C until use for Western blot analysis of proteins. The skeletal muscle tissues were then chopped into pieces before being stored at -80°C. Additionally, the maternal and fetal characteristics were measured, including murine fetal body weights, fetal crown-rump lengths, murine placental weights, and plasma glucose levels.

### Biochemical assays

Serum total cholesterol (TC), triglycerides (TG), high density lipoprotein cholesterol (HDL-C), low density lipoprotein cholesterol (LDL-C), and hepatic levels of cholesterol (TC), triglycerides (TG) were determined, respectively, using enzymatic kits (Nanjing Jiancheng Bioengineering Institute, Jiangsu, China).

### Protein extraction and Western blotting

Murine livers and skeletal muscles were homogenized in ice-cold lysis buffer. After being homogenized on ice for 30 min, the tissue homogenates were centrifuged at 15,000 rpm for 15 min at 4°C. Bicinchoninic acid (BCA) method (Thermo scientific, Rockford, IL, USA) was used for the determination of total protein concentration. The equal amounts of proteins for each lane were aliquoted, denatured and then frozen at -80°C before Western blotting. The proteins were then subjected to sodium dodecyl sulfate polyacrylamide gel electrophoresis (SDS-PAGE) and transferred onto PVDF membranes. After incubation with appropriate primary antibody at 4°C overnight and secondary antibodies at room temperature for 1–3h, polyvinylidene fluoride (PVDF) membranes were washed three times in Tris-buffered saline-T (TBST) for 10min/each and targeted proteins were detected using enhanced chemifluorescence reagent. Obtained bands were quantified using Image Software. Specifically, the membranes were incubated with antiserum against AKT (1:1000, #4691S, Cell Signaling Technology Inc, USA), FoxO1 (1:1000, #2880P, Cell Signaling Technology Inc, USA), PEPCK (1:1000, #12940S, Cell Signaling Technology Inc, USA), G6Pase (1:1000, #RJ2284392, Thermo Scientific, USA), phosphor-AKT T308 (1:1000, #13038P, Cell Signaling Technology Inc, USA), phosphor-AKT S473 (1:1000, #4060P, Cell Signaling Technology Inc, USA), phosphor-FoxO1 (1:1000, #9464P, Cell Signaling Technology Inc, USA), SREBP2 (1:1000, #ab155017, Abcam, USA), ACC (1:1000, #3676P, Cell Signaling Technology Inc, USA), phosphor-ACC (1:1000, #11818S, Cell Signaling Technology Inc, USA), GSK3β (1:1000, #12456P, Cell Signaling Technology Inc, USA) and phosphor-GSK3β (1:1000, #5558P, Cell Signaling Technology Inc, USA), respectively. Each sample was analyzed an average of three independent tests involving different gels.

### Real-time polymerase chain reaction (RT-PCR)

Total RNA was extracted from 50 mg of liver tissues using the Trizol reagent (ThermoFisher, USA) according to the manufacture’s protocol. The concentration and purity of RNA was assessed by NanoDrop ® ND-3000 spectrophotometry (ThermoFisher, USA) at 260 nm and 280 nm. A total of 1μg RNA was used for cDNA synthesis with the PrimeScript RT Reagent Kit Perfect Real Time (TaKaRa, China). PCR amplification was performed with SYBR Premix ExTaq TM II(TaKaRa, China) according to the manufacturer’s instructions. Primers for real-time PCR were designed and synthesized by Invitrogen (Shanghai, China). The primer sequences were as follows: PEPCK (Forward: 5’-CTG GCA CCT CAG TGA AGA CA-3’, Reverse: 5’-TCG ATG CCT TCC CAG TAA AC-3’); G6Pase (Forward: 5’-TTC TGG ATG GTT CCC TGA AG-3’, Reverse: 5’-ACC GCA AGA GCA TTC TCA GT-3’); β-actin (Forward: 5’-CTC TCC CTC ACG CCA TC-3’, Reverse: 5’-ACG CAC GAT TTC CCT TC-3’). Each sample was performed in triplicate in a 25μl reaction volume and β-actin was used as the reference gene. Relative quantification of gene expression was calculated by 2 ^− ΔΔCt^ method based on Ct values for both target and control genes.

### Statistical analysis

All the values were expressed as mean ± standard error of the mean (SEM). For comparisons among several groups, analysis of variance (ANOVA) was initially used. The effects of plasma glucose levels before and after intervention were analyzed by repeated measures with two-way ANOVA followed by Tukey’s multiple comparison test. The effects of fetal body weights, crown-rump lengths, placental weights and all other measured parameters were compared using regular one-way ANOVA and the Student-Newmann-Keuls post hoc test.

## Results

### GDM prompts the hyperglycemia, dampens the growth of pups, but swimming plus metformin significantly improves these characteristics

**Fig 1:**
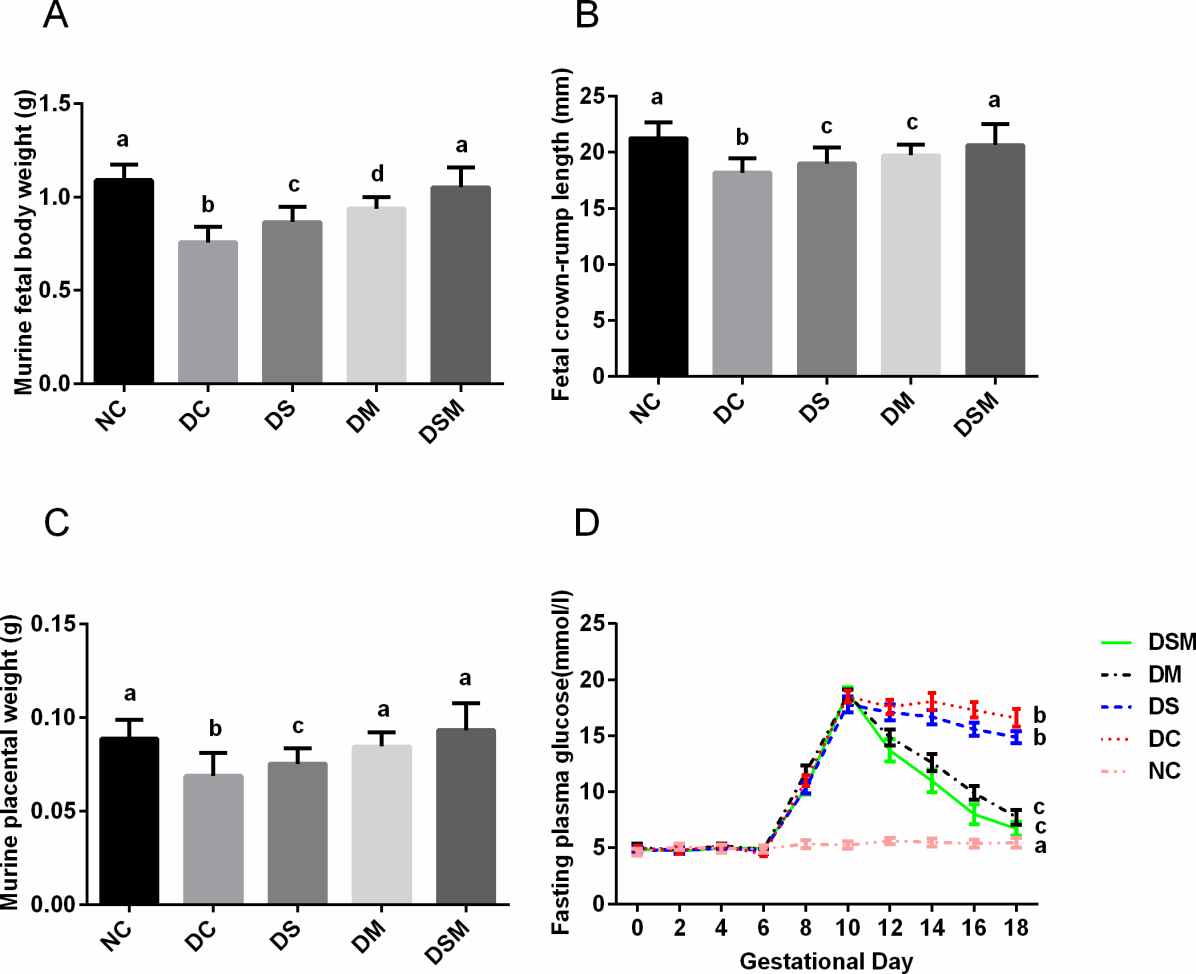
Combined intervention by swimming plus metformin most effectively normalized fetal growth restriction and hyperglycemia caused by GDM. (A) Fetal body weights in different groups with/without interventions. (B) Fetal crown-rump lengths in different groups with/without interventions. (C) Placental weights in different groups with/without interventions. (D) Maternal glucose concentrations from GD0 to GD18 in different groups with/without interventions. The blood glucose values among different groups in this study are compared in GD18. The values represent the mean ± SEM, n = 10/group. The columns with different letter (a, b, c, d) are significantly different, *P*<0.05 (ANOVA, SNK). NC: normal control group, DC: GDM control group, DS: GDM treated with swimming group, DM: GDM treated with metformin group, DSM: GDM treated with swimming plus metformin group.

The growth of fetal mice was generally normal in normal control group. Compared with the NC group, GDM fetuses obtained significant decreases in the body weight, crown-rump length and placental weight (Fig 1). The birth weight of pups in the DS, DM and DSM groups were (0.866±0.011)g, (0.938±0.008)g and (1.052±0.012)g, respectively, bigger than the DC group (0.756±0.011)g (*P*<0.05), but still smaller than the NC group (1.089±0.011)g (*P*<0.05, Fig 1A). The final placental weight of mice and crown-rump length of pups among all groups were not significantly differed from NC group but the values in DC group mice were the lowest (*P*<0.05, Fig 1B and 1C). These characteristic differences were mostly corrected by combined action of metformin plus swimming. Compared with the DC group, the mothers of swimming-treated group had just mild decrease of plasma glucose levels, which is not statistically significant, while metformin-treated group had significantly lower levels of plasma glucose (*P*<0.05), but still higher than NC group (*P*<0.05). After combined intervention, although plasma glucose levels of DSM group were higher than the NC group (*P*<0.05), which was similar to DM group, the overall inhibition of plasma glucose levels were the most effective than those in other groups (*P*<0.05, Fig 1D. Note: The blood glucose values among different groups in this study are compared in GD18). Furthermore, the body weight of the pregnant mice are displayed in the S1 Table. The weight of pregnant mice in DC group was significantly lower than that in NC group, and returned to normal level after metformin intervention and combined intervention.

### Swimming plus metformin intervention sharply ameliorates lipid metabolism in GDM

**Fig 2:**
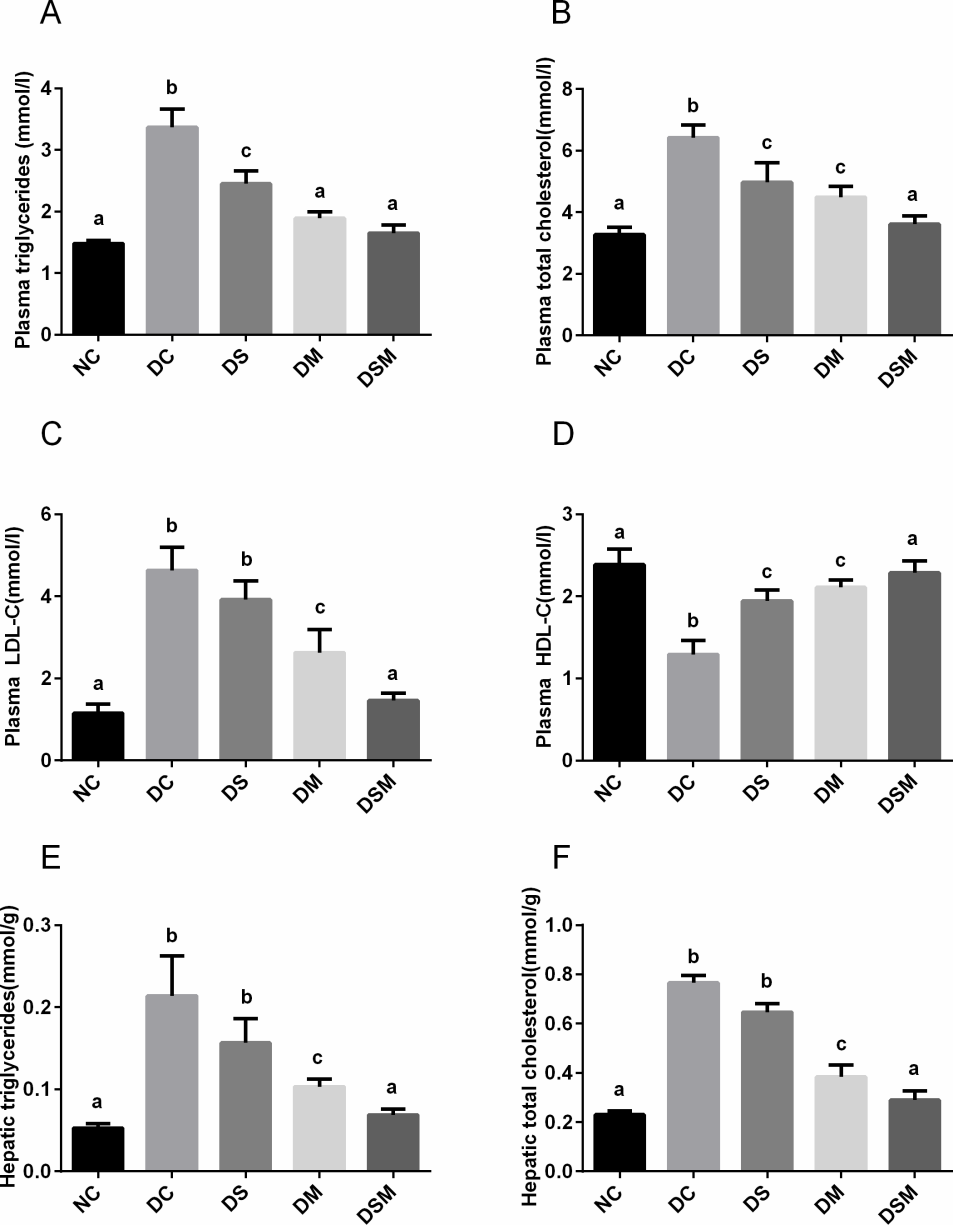
Swimming plus metformin intervention sharply ameliorates lipid metabolism in GDM. (A) Murine plasma TG levels in different groups. (B) Murine plasma TC levels in different groups. (C) Murine plasma LDL-C levels in different groups. (D) Murine plasma HDL-C levels in different groups. (E) Effects of various interventions on TG levels in the murine livers. (F) Effects of various interventions on TC levels in the murine livers. The values represent the mean ± SEM, n = 10/group. The columns with different letter (a, b, c) are significantly different, *P*<0.05 (ANOVA, SNK). All data in this figure are representatives of three independent experiments. NC: normal control group, DC: GDM control group, DS: GDM treated with swimming group, DM: GDM treated with metformin group, DSM: GDM treated with swimming plus metformin group.

The lipid profiles of each group are shown in Fig 2. Serum levels of TC, LDL-C, and TG were markedly elevated while HDL-C was decreased in the DC group compared with the NC group (*P*<0.05). Compared with the DC group, the pregnant mice in swimming-treated group and metformin-treated group had lower levels of TG and TC (*P*<0.05), which were still higher than those in NC group (*P*<0.05). The changing trend of HDL-C was just opposite in the two groups. Importantly, those effects were ameliorated significantly by the swimming plus metformin treatment in DSM group (*P*<0.05), in which the levels of four biochemical indexes were close to NC group (Fig 2A–2D). In addition, the levels of TC and TG in the livers were also detected, consistent with the trends in plasma (Fig 2E and 2F).

### Intervention by swimming plus metformin normalizes the hepatic insulin sensitivity under GDM condition

**Fig 3:**
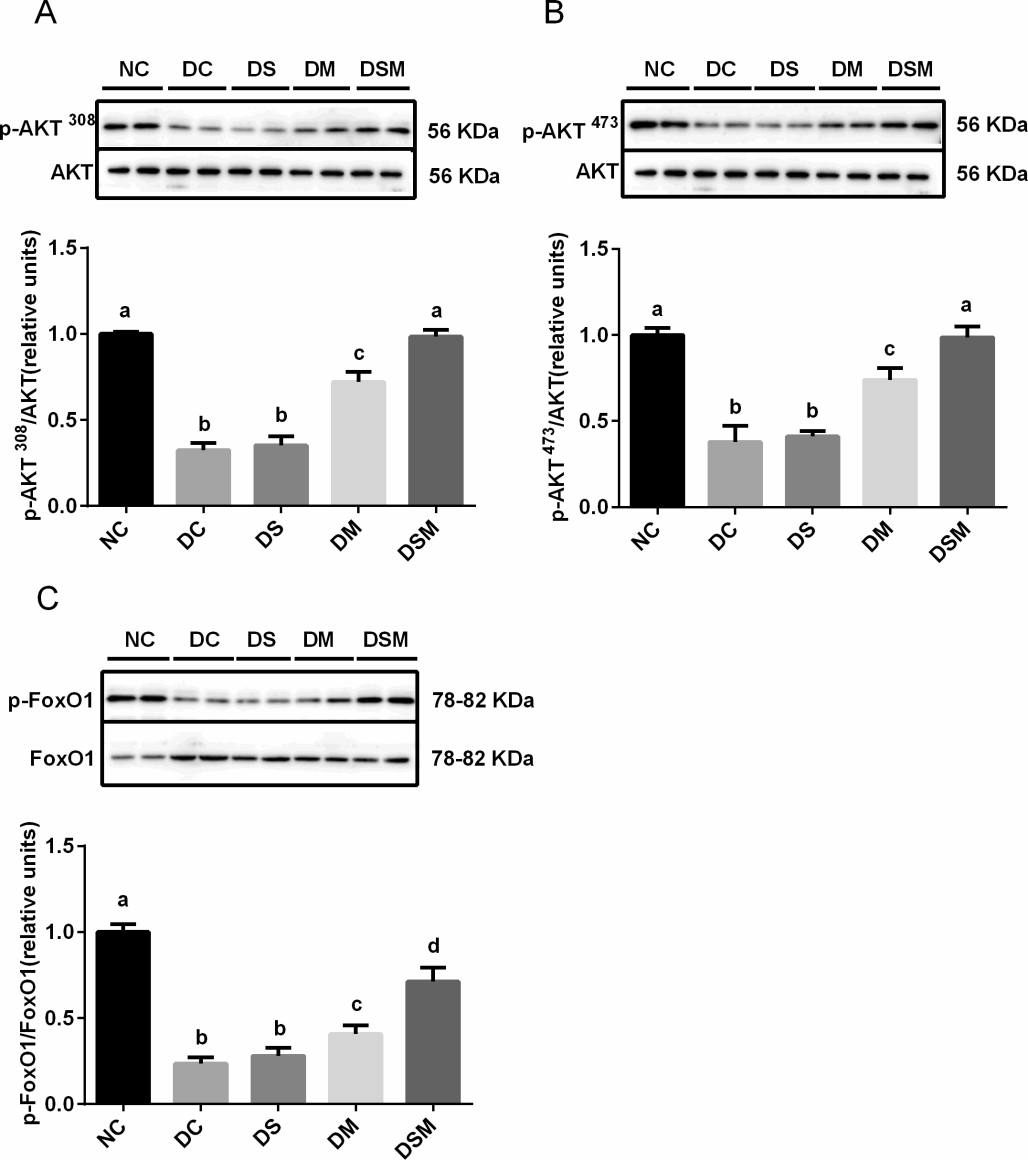
Intervention by swimming plus metformin normalizes the hepatic insulin sensitivity under GDM condition. Levels of AKT Thr308 phosphorylation (p-AKT T308) were normalized to total AKT in the livers treated with swimming or metformin or both combined. (B) Levels of phosphorylated serine-473 AKT (p-AKT S473) were normalized to total AKT in the livers treated with swimming or metformin or both combined. (C) Levels of phosphorylated FoxO1 were normalized to total FoxO1 in the livers intervened by swimming or metformin or both combined. The values represent the mean ± SEM, n = 10/group. The columns with different letter (a, b, c, d) are significantly different, *P*<0.05 (ANOVA, SNK). All data in this figure are representatives of three independent experiments. NC: normal control group, DC: GDM control group, DS: GDM treated with swimming group, DM: GDM treated with metformin group, DSM: GDM treated with swimming plus metformin group.

To understand the mechanism by which intervention enhanced insulin sensitivity, we examined the phosphorylation levels of AKT (Thr308) and AKT (Ser473) as well as the phosphorylation level of p-FoxO1. We found that compared with DC group, intervention by swimming alone did not improve the AKT activity, while intervention by metformin alone significantly ameliorated AKT phosphorylation. Of note, compared to the swimming alone or metformin alone, the combined therapy by swimming plus metformin most effectively enhanced AKT activity (p-AKT S473 and p-AKT T308), which was close to that in NC group. Additionally, due to the total AKT protein levels in maternal liver tissues being not obviously changed in the four GDM groups compared to the NC group, the ratios of p-AKT S473 and p-AKT T308 to total AKT were significantly decreased in DC group (*P*<0.05). Furthermore, DS group showed no significant change of the ratios compared with DC group, but those in DM group were elevated (*P*<0.05). Meanwhile, the DSM group displayed tremendously recovered activity of AKT (both p-AKT S473 and p-AKT T308) in maternal livers (Fig 3A and 3B).

The same pattern of effects on FoxO1 phosphorylation by various interventions was also observed in GDM mice. In addition, the total protein levels of FoxO1 were significantly higher in the four GDM groups than the normal control group (*P*<0.05). The ratios of p-FoxO1 to FoxO1 were significantly decreased in DC group, but elevated dramatically by the combined action of swimming plus metformin supplementation (*P*<0.05, Fig 3C). These results indicated that combined interventions enormously improved insulin signaling.

### Combined intervention ameliorates the hepatic de novo lipogenesis under GDM condition

**Fig 4:**
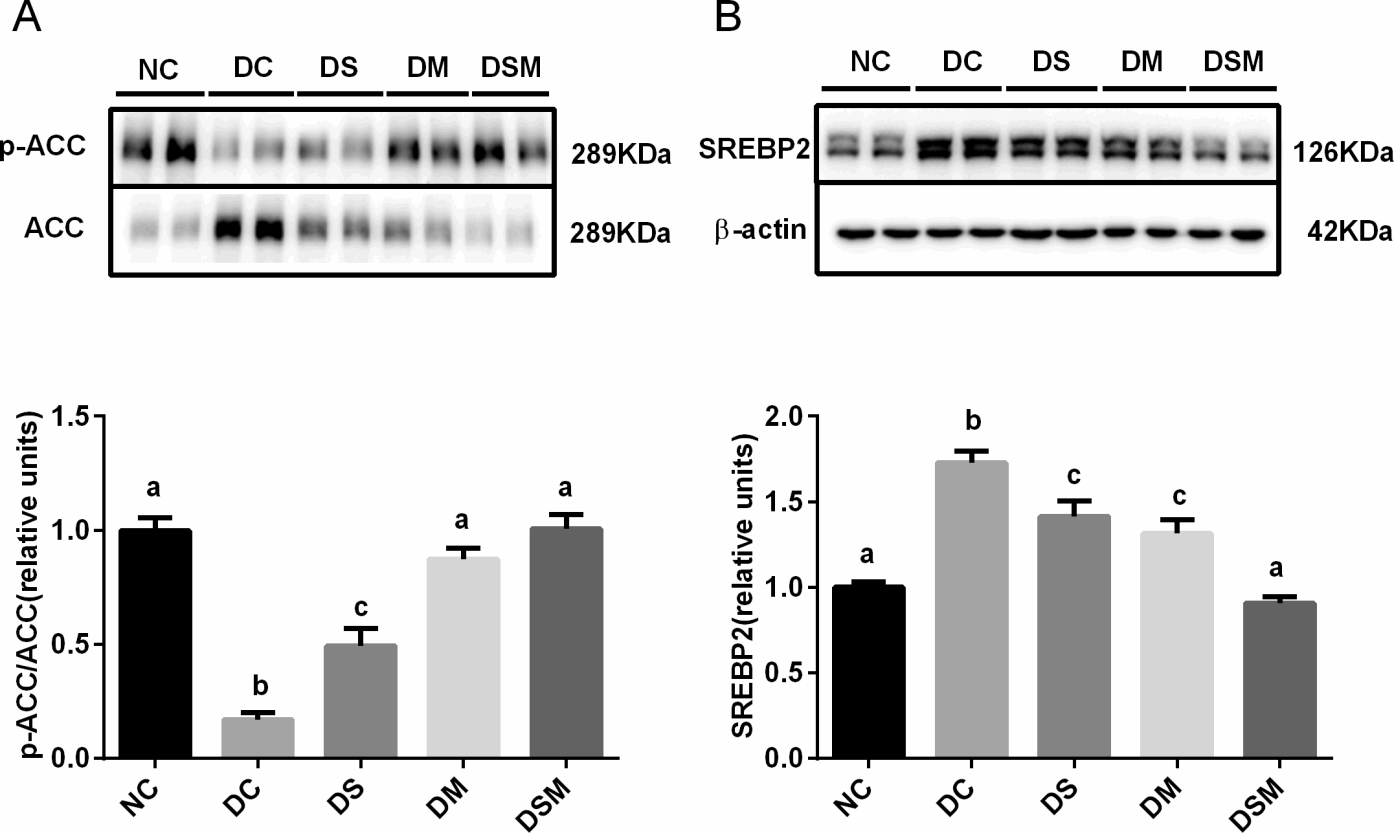
Combined intervention of swimming plus metformin ameliorates the hepatic *de novo* lipogenesis under GDM condition. (A) Levels of phosphorylated ACC were normalized to total ACC in the livers treated with swimming or metformin or both combined. (B) Expression of SREBP2 were normalized to β-actin in the livers intervened by swimming or metformin or both combined. The values represent the mean ± SEM, n = 10/group. The columns with different letter (a, b, c) are significantly different, *P*<0.05 (ANOVA, SNK). All data in this figure are representatives of three independent experiments. NC: normal control group, DC: GDM control group, DS: GDM treated with swimming group, DM: GDM treated with metformin group, DSM: GDM treated with swimming plus metformin group.

We investigated whether the altered metabolic phenotypes of the GDM maternal were the consequences of altered the protein expression of enzymes and transcription factors involved in hepatic lipid metabolism. Our immunoblots showed that total ACC protein levels were markedly increased in the livers of GDM mothers (*P*<0.05), swimming or metformin can effectively reduce the protein expression of ACC in moderate degrees, and ultimately, the DSM group showed most effective suppression of total ACC proteins, which was totally identical to NC group. Thereby, the ratios of p-ACC to ACC were significantly decreased in the DC and DS groups compared to the normal control group (*P*<0.05). Those ratios in DM group were moderately elevated compared to the DC group (*P*<0.05). While the ratios in DSM group were showed dramatic elevation compared to DC and DS groups, and no significant change compared with NC group, suggesting that combined intervention may most effectively and substantially inhibit the activity of ACC, a key enzyme required for fatty acid synthesis (Fig 4A).

Moreover, the protein levels of SREBP2 in liver tissues were greatly induced by GDM compared to the normal control group, but this induction was totally suppressed by the combined action of swimming plus metformin (*P*<0.05, Fig 4B).

### Combined intervention suppresses hepatic gluconeogenesis under GDM condition

**Fig 5:**
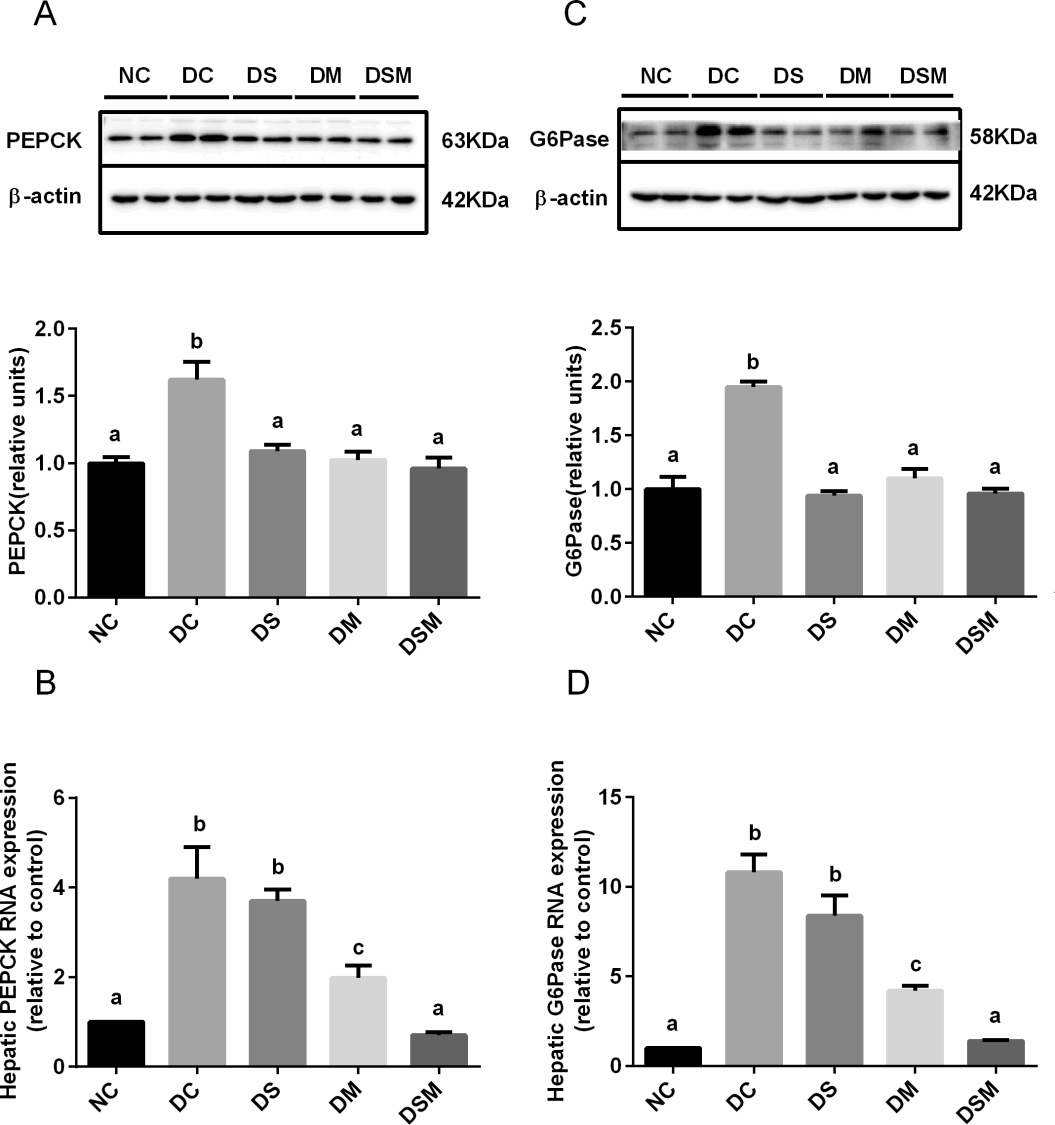
Combined intervention of swimming plus metformin suppresses hepatic gluconeogenesis under GDM condition. (A) Protein expression of PEPCK in the livers from the mice in NC, DC, DS, DM and DSM groups, respectively. Results were normalized to β-actin and the fold change was calculated relative to DC group. (B) mRNA levels of PEPCK in the same livers in (A). (C) Protein expression of G6Pase in the livers from the mice in NC, DC, DS, DM and DSM groups, respectively. Results were normalized to β-actin and the fold change was calculated relative to DC group. (D) mRNA levels of G6Pase in the same livers in (C). mRNA levels were normalized to β-actin. The values represent the mean ± SEM, n = 10/group. The columns with different letter (a, b, c) are significantly different, *P*<0.05 (ANOVA, SNK). All data in this figure are representatives of three independent experiments. NC: normal control group, DC: GDM control group, DS: GDM treated with swimming group, DM: GDM treated with metformin group, DSM: GDM treated with swimming plus metformin group.

PEPCK and G6Pase catalyze committed steps of gluconeogenesis, thus play crucial roles in glucose homeostasis. We used the western blotting and real-time PCR to assess the effects of different interventions on the tow gene expressions in GDM livers. The gene expressions of PEPCK and G6Pase were significantly increased in livers of DC group, respectively, compared to NC group (*P*<0.05). But the elevations were sharply dampened by all kinds of interventions (Fig 5A). In addition, mRNA levels of PEPCK and G6Pase declined in intervention groups, particularly in combined intervention group, compared to DC group (*P*<0.05, Fig 5C and 5D). Moreover, the G6Pase gene expression was more sensitive to interventions, especially to combined intervention (Fig 5B).

### Combined intervention improves the insulin signaling in skeletal muscle tissues under GDM condition

**Fig 6:**
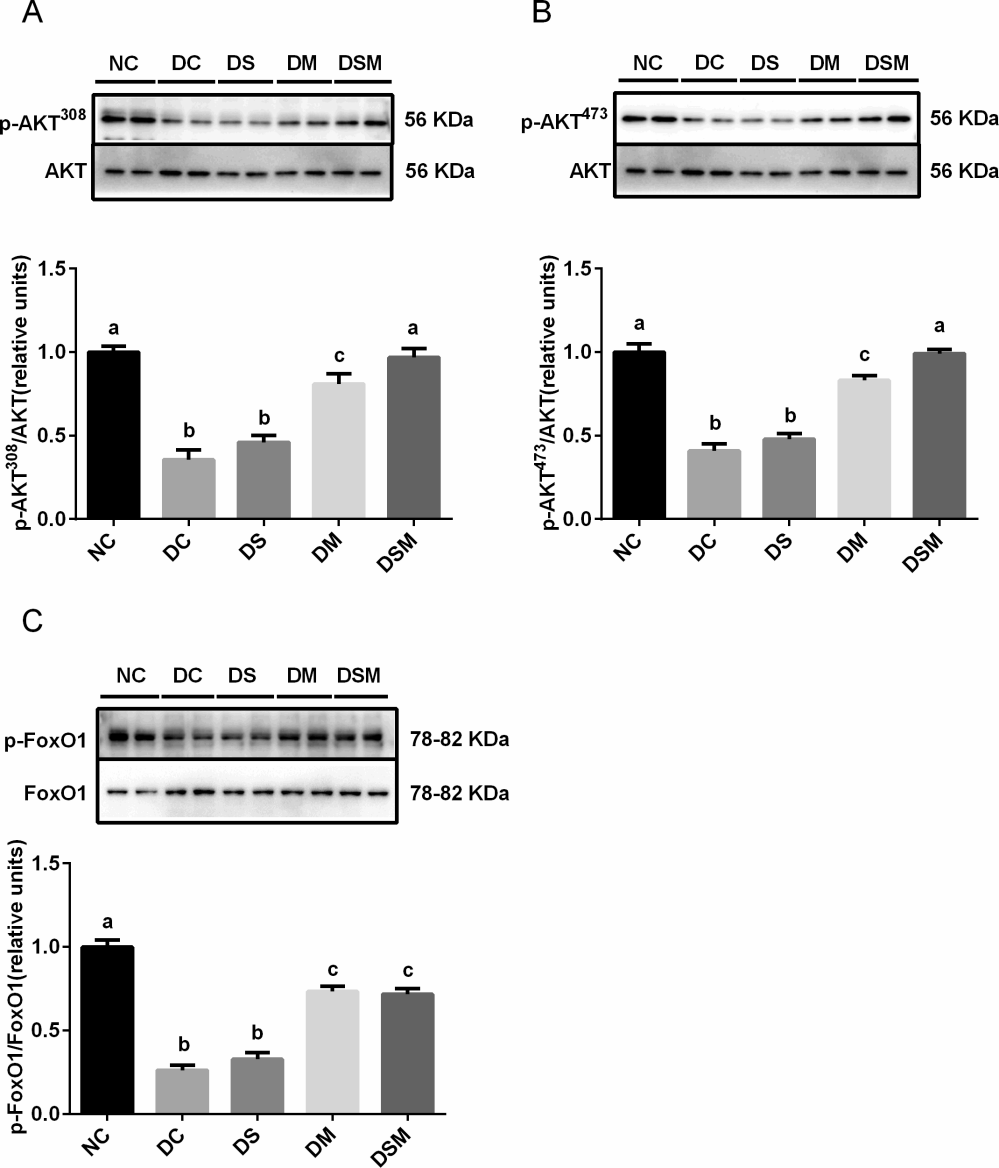
Combined intervention of swimming plus metformin improves the insulin signaling in skeletal muscle tissues under GDM condition. Thr308 phosphorylation levels of AKT (p-AKT T308) were normalized to total AKT in the skeletal muscle treated with swimming or metformin or both combined. (B) Levels of phosphorylated serine-473 AKT (p-AKT S473) were normalized to total AKT in the skeletal muscle treated with swimming or metformin or both combined. (C) Levels of phosphorylated FoxO1 were normalized to total FoxO1 in the skeletal muscle intervened by swimming or metformin or both combined. The values represent the mean ± SEM, n = 10/group. The columns with different letter (a, b, c) are significantly different, *P*<0.05 (ANOVA, SNK). All data in this figure are representatives of three independent experiments. NC: normal control group, DC: GDM control group, DS: GDM treated with swimming group, DM: GDM treated with metformin group, DSM: GDM treated with swimming plus metformin group.

Fig 6A and 6B demonstrate the effects of swimming or metformin or both combined on the phosphorylation of AKT in skeletal muscle. The levels of total AKT proteins in maternal skeletal muscle tissues were sharply increased in DC group, while those were not obviously changed in the GDM-intervened groups compared to the normal control group. Levels of p-AKT S473 and p-AKT T308 were significantly decreased in DC group, and the decrease was substantially rescued by the combined action of swimming plus metformin.

In addition, the protein levels of FoxO1 in skeletal muscle tissues were also higher in GDM groups than those in NC group. The ratios of p-FoxO1 to FoxO1 were significantly decreased both in DC and DS groups compared to the normal control group (Fig 6C), suggesting that swimming alone cannot improve the insulin sensitivity in GDM skeletal muscle. However the ratios in DM group were significantly elevated compared to DC group. Meanwhile, the ratios in DSM group were showed strongest increase than NC and other groups, indicating that the combined intervention dramatically improves the insulin sensitivity in skeletal muscle under GDM condition (Fig 6C).

### Combined intervention boosts the energy storage and improves de novo lipogenesis in GDM skeletal muscle

**Fig 7:**
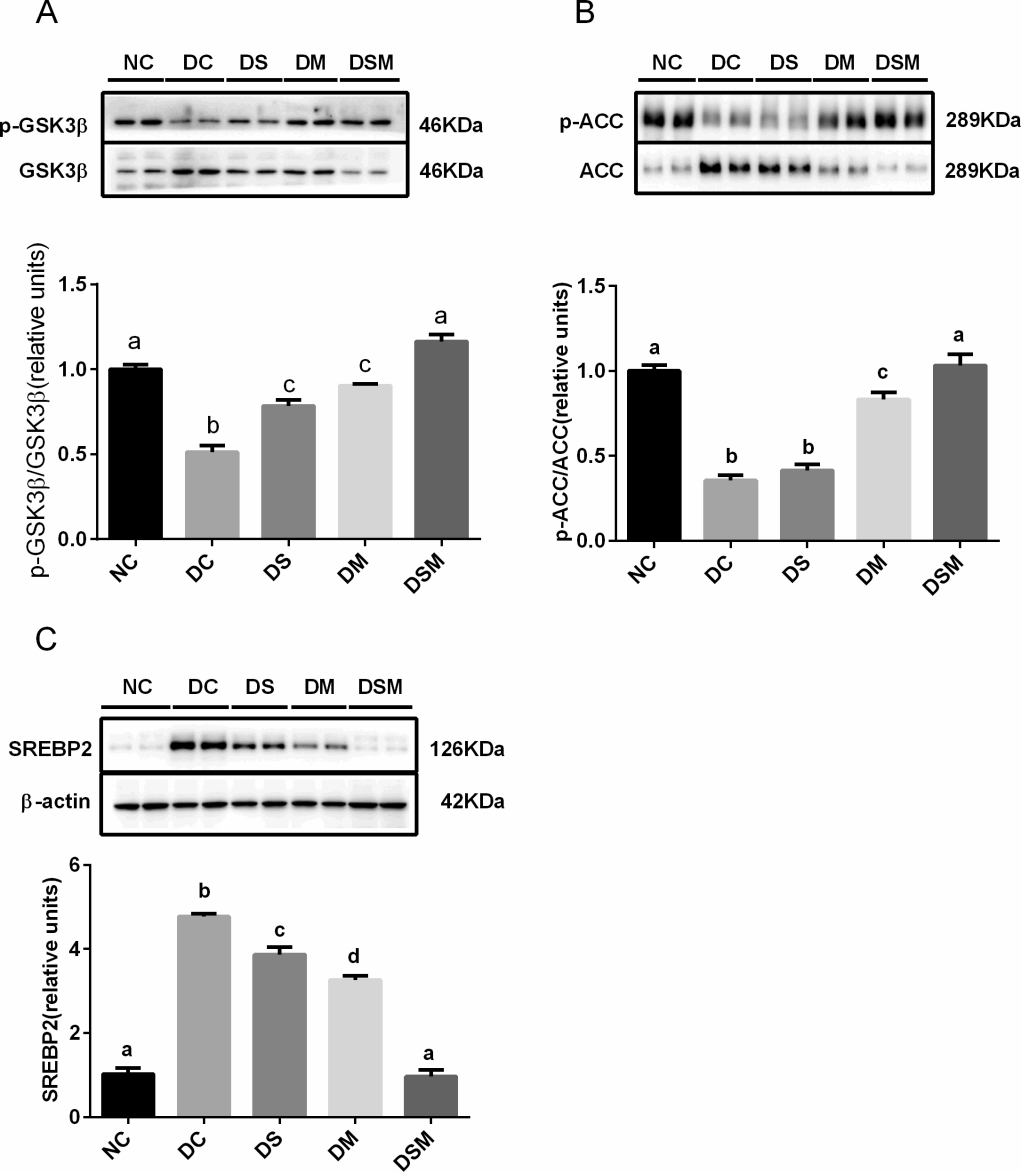
Combined intervention of swimming plus metformin boosts the energy storage and improves de novo lipogenesis in GDM skeletal muscle tissues. (A) Phosphorylation of GSK3β was analyzed by immunoblotting and normalized to total GSK3β proteins in skeletal muscle treated with swimming or metformin or both combined. (B) Levels of phosphorylated ACC in different groups were normalized to total ACC in the skeletal muscle treated with swimming or metformin or both combined. (C) Expression of SREBP2 was normalized to β-actin in the skeletal muscle treated with swimming or metformin or both combined. The values represent the mean ± SEM, n = 10/group. The columns with different letter (a, b, c, d) are significantly different, *P*<0.05 (ANOVA, SNK). All data in this figure are representatives of three independent experiments. NC: normal control group, DC: GDM control group, DS: GDM treated with swimming group, DM: GDM treated with metformin group, DSM: GDM treated with swimming plus metformin group.

Because GSK3β activity plays important role in glycogen synthesis, we examined both phosphorylation levels and protein levels of GSK3β in skeletal muscle tissues. The immunoblots showed that various interventions on GDM significantly increased the levels of inactive form of GSK3β (phosphorylated form) in different degrees while reduced the total GSK3β protein levels compared to DC group. As a result, the ratios of p-GSK3β to total GSK3β in GDM groups were greatly enhanced by all kinds of interventions, among which combined intervention showed most effective elevation. These data suggest that combined intervention may be the best way to promote the energy storage (glucose conversion to glycogen) in skeletal muscle, thereby ameliorating the hyperglycemia in GDM (Fig 7A).

Fig 7B showed that total ACC proteins were markedly increased in the skeletal muscles from DC and DS groups, and that metformin alone or combined intervention may effectively reduce the expression of total ACC proteins. Of note is that the two interventions, especially combined intervention, tremendously elevated the phosphorylation of ACC (inactivation of ACC), resulting in the ratios of p-ACC to total ACC being dramatically enhanced in DM and DSM groups. These effects indicated that two interventions, especially combined intervention may more effectively inhibit the ACC activity, thereby suppressing its regulated *de novo* lipogenesis in skeletal muscle under GDM condition.

In addition, the protein levels of SREBP2 in skeletal muscle tissues were higher in the GDM groups than the normal control group. The proteins were decreased slightly by swimming alone, and sharply by metformin treatment, while combined intervention totally normalized the SREBP2 protein expression compared to the NC group (Fig 7C), revealing that swimming plus metformin may be the best way to inhibit the cholesterol synthesis in skeletal muscles.

## Discussion

It has been long recognized that GDM is associated with a markedly increased risk of perinatal mortality and morbidity [31–32]. Traditionally, GDM is tightly associated with macrosomia or fetal growth restriction in humans [33–35]. Our results showed that murine GDM may result in intrauterine growth restriction, which is consistent with some findings indicating that exposure to this diabetic intrauterine environment causes inhibition in fetal growth, and predisposes to abnormal glucose tolerance and insulin resistance even in the absence of macrosomia at birth [36–37].

In addition, it has been thought that insulin resistance (IR) is the common pathogenesy of GDM and type 2 diabetes mellitus (T2DM) [38]. In our current study, we did demonstrate that murine GDM mothers had high insulin resistance, resulting in a greater rise of plasma glucose and an abnormal lipid metabolism than those in normal controls. However, the high plasma glucose levels in GDM mothers could be improved through single metformin intervention in a moderate degree, or be totally rescued by combined intervention. While the effect of swimming alone intervention on plasma glucose level was not statistically significant in GDM mice during pregnancy, which could be attributed to the limited times and intensity of swimming performed per day. Compared to the long-term and tense swimming training, our exercise intervention may not be sufficient both in duration and in strength due to the limited intensity allowed for pregnant mice, suggesting that swimming alone may not be good enough as a way of GDM mice treatment [30,39–40]. However, the combined therapy of limited swimming plus metformin in DSM group showed totally recovered insulin sensitivity.

The hypoglycemic activity of metformin is related to the reduction of hepatic gluconeogenesis, and stimulation of glucose utilization in the skeletal muscle [41]. Our current study clearly indicated that metformin may prompt the weak or moderate phosphorylation of AKT at Thr308(p-AKT T308) and Ser473 (p-AKT S473) in the livers and skeletal muscle tissues, while combined therapy totally normalized AKT activity, which demonstrated no significant difference from the NC group, indicating the most effective intervention by superposition effects.

Both phosphoenolpyruvate carboxykinase (PEPCK) and glucose-6- phosphatase (G6Pase) catalyze committed steps of gluconeogenesis, which are regulated by FoxO1 [39,42]. Our current study clearly confirmed that protein levels of PEPCK and G6Pase were significantly increased in GDM groups, leading to elevated hepatic gluconeogenesis. Importantly, our data showed that although three interventions may significantly lower the PEPCK protein levels in GDM mice compared to DC group, only did the combined intervention exert most effective and drastic inhibition of G6Pase levels.

Consistent to previous reports, our current data also indicated that GDM significantly enhances the harmful lipid levels both in plasma and in liver. ACC was reported to provide the malonyl-CoA as a substrate for the biosynthesis of fatty acids [43]. Our data indicated that phosphorylations of ACC in GDM groups were significantly inhibited, while total ACC protein levels were increased, implying that the ACC activity was enhanced in GDM state. Our data also indicated that swimming or metformin may significantly alleviates the ACC activity in GDM groups, and that only the combined intervention totally suppressed the ACC activity compared to NC group.

SREBP2 plays a vital role in the regulation of cholesterol synthesis, and AKT is implicated in the regulation of lipid metabolism through the activation of SREBP2 [44–45]. In the present study, we found that swimming or metformin alone may significantly ameliorate the SREBP2 protein levels in livers and skeletal muscle tissues from GDM mice, and that only combined therapy of swimming plus metformin totally suppressed SREBP levels in both organs compared to the NC group, suggesting that the combined intervention may be the most effective therapy in correcting the dysregulation of cholesterol synthesis both in liver and skeletal muscle from GDM mice.

Glycogen synthase kinase 3 (GSK3) α and β are serine/threonine protein kinases that are involved in the storage of glucose in a form of glycogen both in liver and skeletal muscle. It is well known that inhibition of AKT phosphorylation downregulates GSK3β phosphorylation, thereby inhibiting glycogen synthase (GS) activity [46]. The previous study suggested that GSK3β activity be increased in skeletal muscle samples from subjects with insulin resistance [47] and that phosphorylated (inactive) GSK3β expression was lowered in skeletal muscle obtained from women with GDM [7], which are consistent with our results. More importantly, our current data indicated that although swimming alone may slightly, or metformin may moderately inhibit the GSK3β activity *via* increasing its phosphorylation level, only did the combined intervention elicit most effective inhibition of GSK3β activity or promotion of GS activity, thereby alleviating the hyperglycemia in GDM mice through boosting the energy storage or glucose conversion to glycogen in skeletal muscle.

Taken together, our experimental data clearly showed that GDM exerts a strong effect on the subsequent development of insulin resistance and lipid disorder in murine GDM mothers. The combined intervention by metformin plus swimming may be more effective than any single action to ameliorate glucose and lipid metabolism *via* improving insulin sensitivity both in liver and skeletal muscle in GDM mice.

## Supporting information

S1 Table Changes in weight of GDM pregnant mice. (DOC)

S1 File Raw data for Fig 1. (XLS)

S2 File Raw data for Fig 2. (XLS)

S3 File Raw data for Fig 3. (DOC)

S4 File Raw data for Fig 4. (DOC)

S5 File Raw data for Fig 5. (DOC)

S6 File Raw data for Fig 6. (DOC)

S7 File Raw data for Fig 7. (DOC)
