# Supplementary material for: Combined intervention of swimming plus metformin ameliorates the insulin resistance and impaired lipid metabolism in murine gestational diabetes mellitus
Source: PLoS One. 2018 Apr 20;13(4):e0195609. doi: 10.1371/journal.pone.0195609 (PMC5909919; doi:10.1371/journal.pone.0195609)
Supplement: S1 Table — (DOC) [file pone.0195609.s001.doc]

**S1 Table**. Changes in weight of GDM pregnant mice

|  | **NC** | **DC** | **DS** | **DM** | **DSM** |
| --- | --- | --- | --- | --- | --- |
| **GD0** | 18.96±0.31 | 19.26±0.22 | 19.67±0.23 | 19.58±0.28 | 19.71±0.23 |
| **GD2** | 20.38±0.33 | 19.86±0.42 | 20.22±0.27 | 20.46±0.20 | 20.44±0.22 |
| **GD4** | 22.05±0.37 | 21.02±0.34 | 21.04±0.31 | 21.55±0.28 | 21.34±0.26 |
| **GD6** | 23.07±0.43 | 21.99±0.59 | 22.60±0.45 | 22.83±0.31 | 22.50±0.30 |
| **GD8** | 24.62±0.53 | 22.13±0.78^**^ | 22.79±0.30 | 23.60±0.26 | 23.85±0.38 |
| **GD10** | 26.00±0.61 | 23.50±0.82^**^ | 23.98±0.22 | 25.08±0.29 | 25.29±0.36 |
| **GD12** | 27.22±0.79 | 25.04±0.93^*^ | 25.20±0.32 | 26.48±0.28 | 26.67±0.38 |
| **GD14** | 28.74±0.79 | 26.43±0.85^*^ | 26.60±038 | 28.09±0.34 | 28.31±0.49 |
| **GD16** | 30.19±0.81 | 27.79±0.88^**^ | 28.14±0.50 | 29.46±0.41 | 29.86±0.55^#^ |
| **GD18** | 31.66±0.87 | 28.87±0.97^**^ | 29.64±0.52 | 30.99±0.44^#^ | 31.38±0.61^##^ |

The values represent the mean±SEM, n=10/group. **P*<0.05 vs NC, ***P*<0.01 vs NC; # *P*<0.05 vs DC, ## *P*<0.01 vs DC (ANOVA, LSD). NC: normal control group, DC: GDM control group, DS: GDM treated with swimming group, DM: GDM treated with metformin group, DSM: GDM treated with swimming plus metformin group.
