# Supplementary figures and images for: Combined intervention of swimming plus metformin ameliorates the insulin resistance and impaired lipid metabolism in murine gestational diabetes mellitus
Source: PLoS One. 2018 Apr 20;13(4):e0195609. doi: 10.1371/journal.pone.0195609 (PMC5909919; doi:10.1371/journal.pone.0195609)

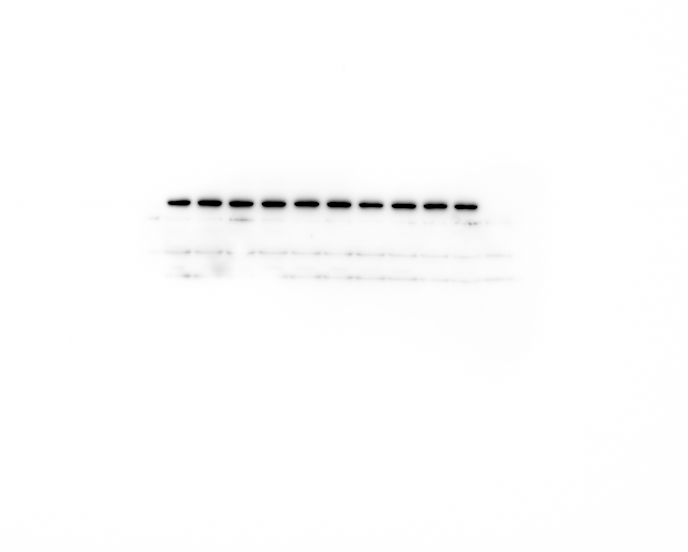


**AKT**


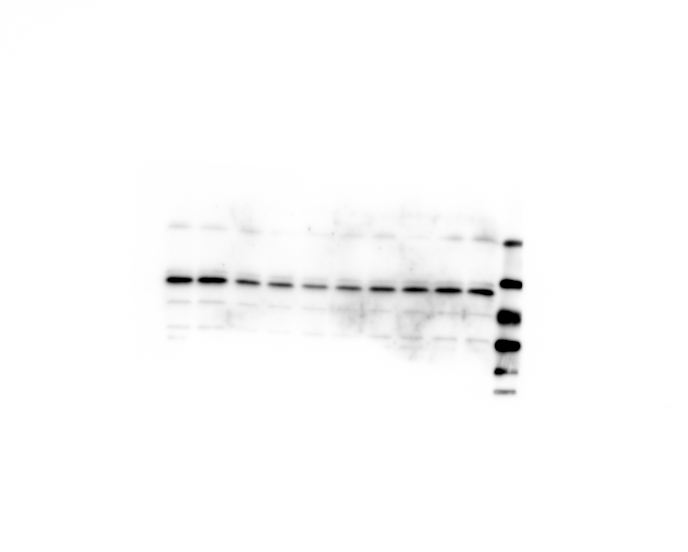


**p-AKT(308)**


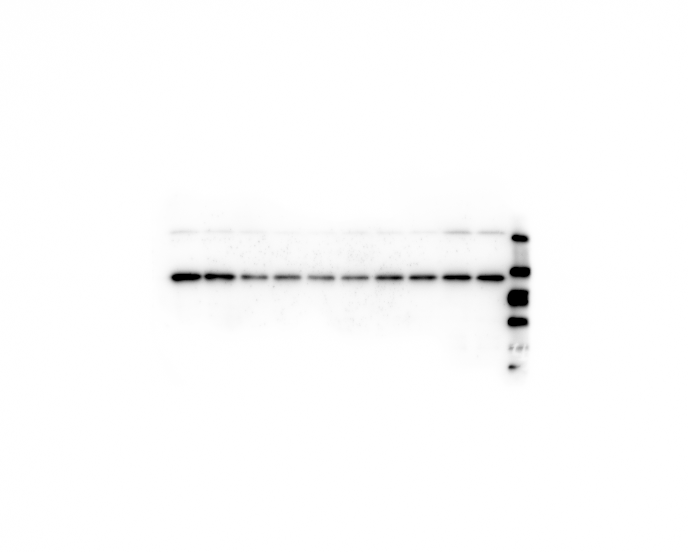


**p-AKT(473)**

[
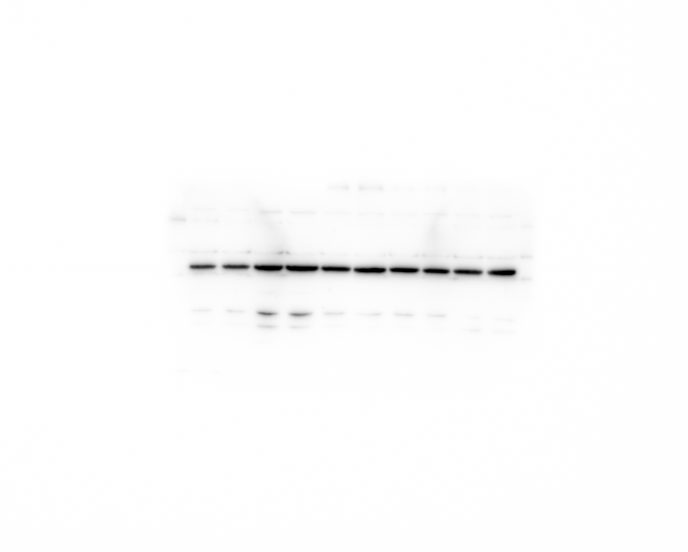
](file:///tmp/out/work/.ptmp6216/Data%20Review/FoxO1.tiff.tif)

**FoxO1**


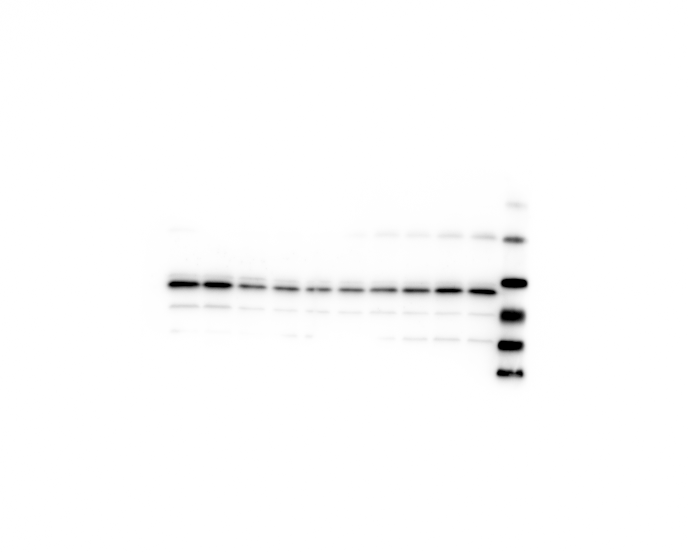


**p-FoxO1**

Supplement: S3 File — (DOC) [file pone.0195609.s004.doc]

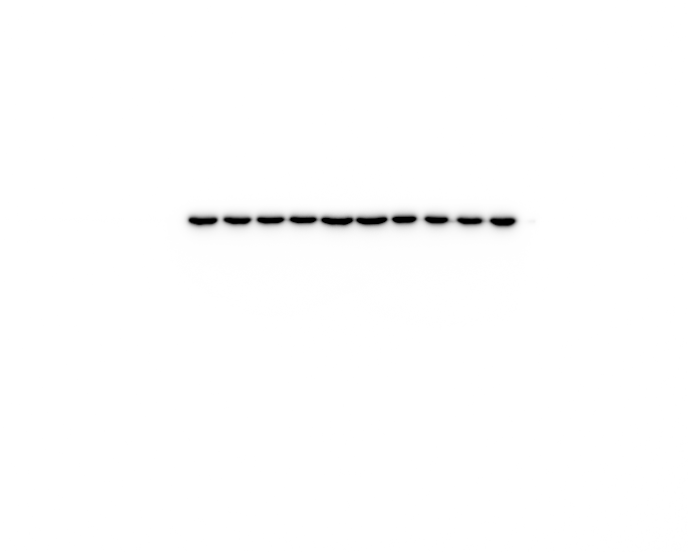


**β-actin**


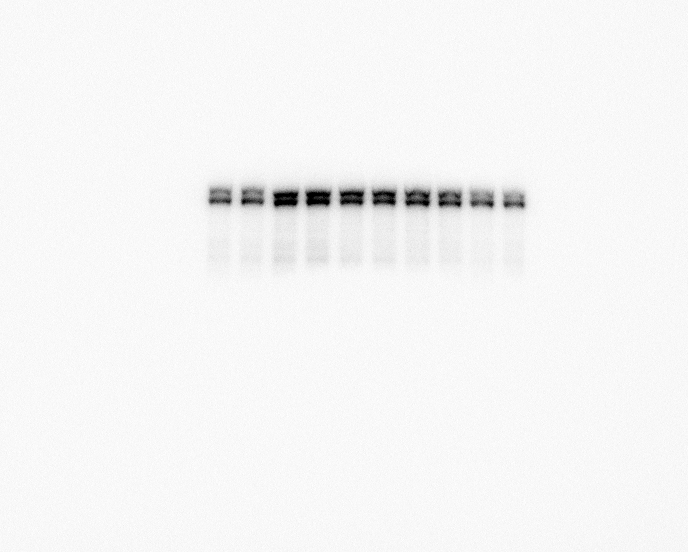


**SREBP2**


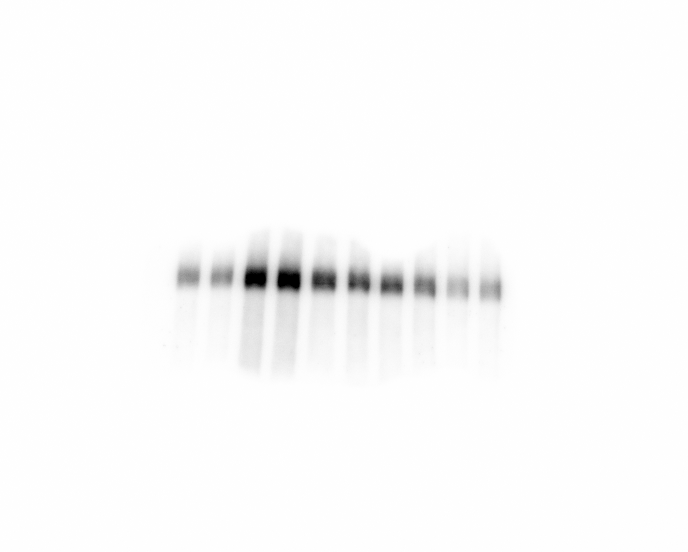


**ACC**


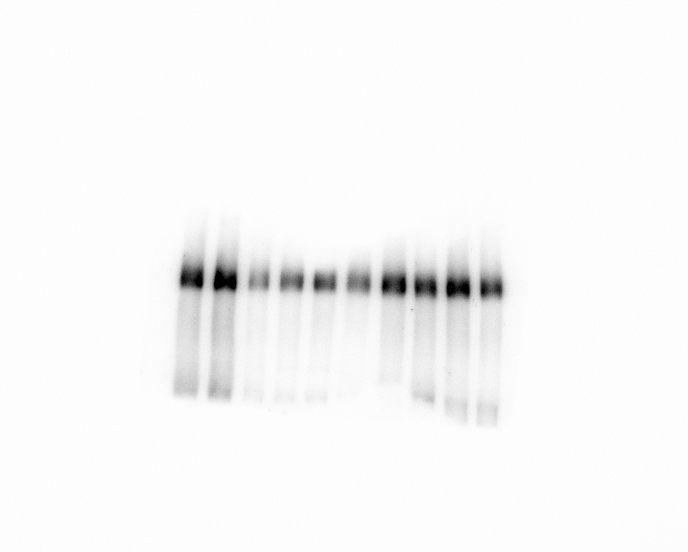


**p-ACC**

Supplement: S4 File — (DOC) [file pone.0195609.s005.doc]

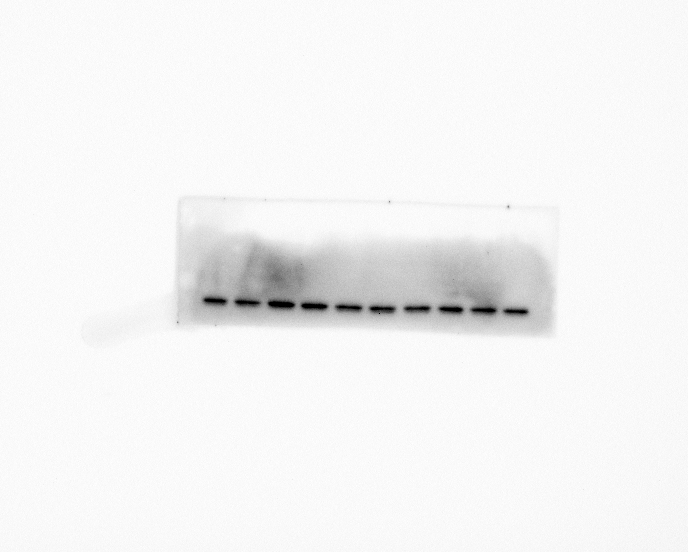


AKT


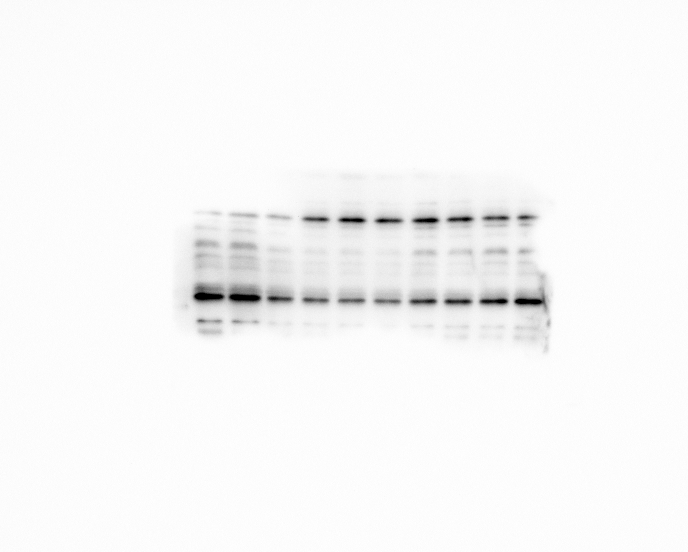


p-AKT(308)


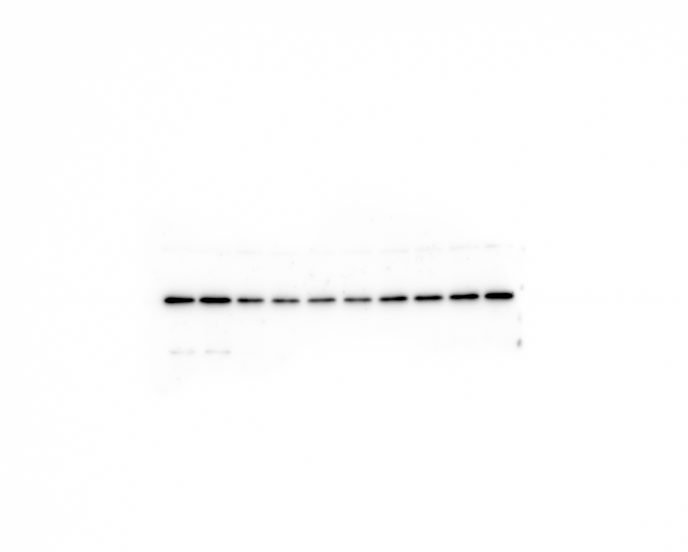


p-AKT(473)


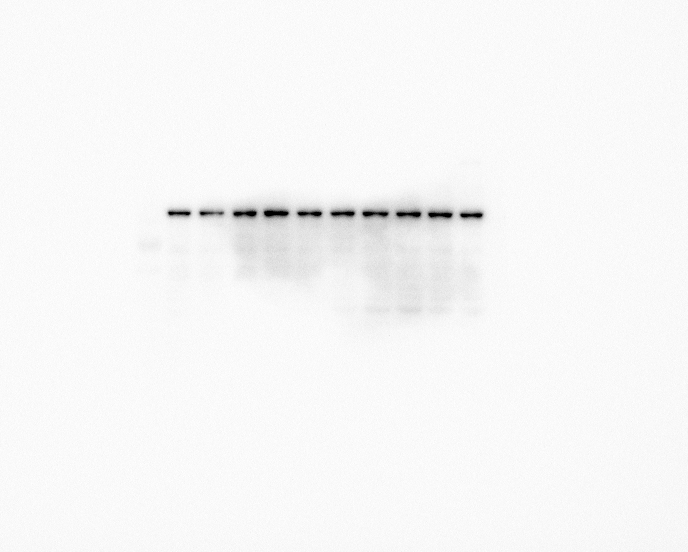


FoxO1


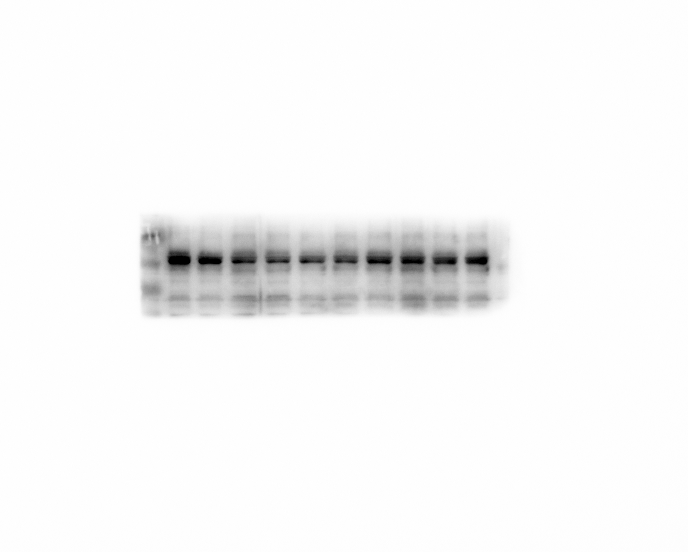


p-FoxO1

Supplement: S6 File — (DOC) [file pone.0195609.s007.doc]

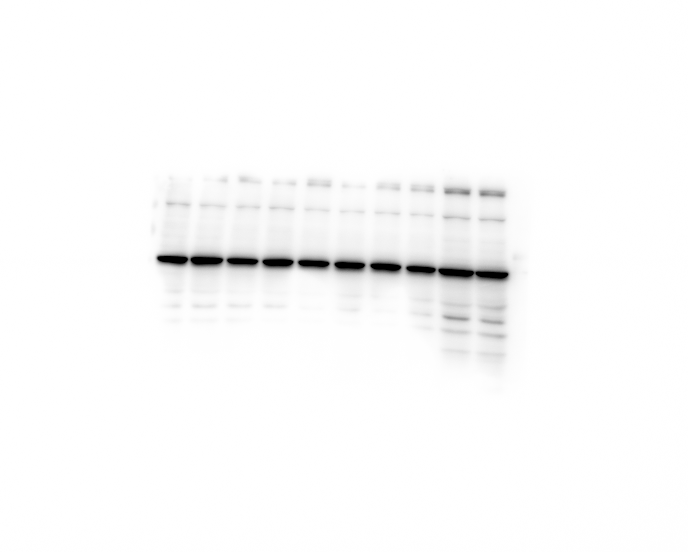


β-actin


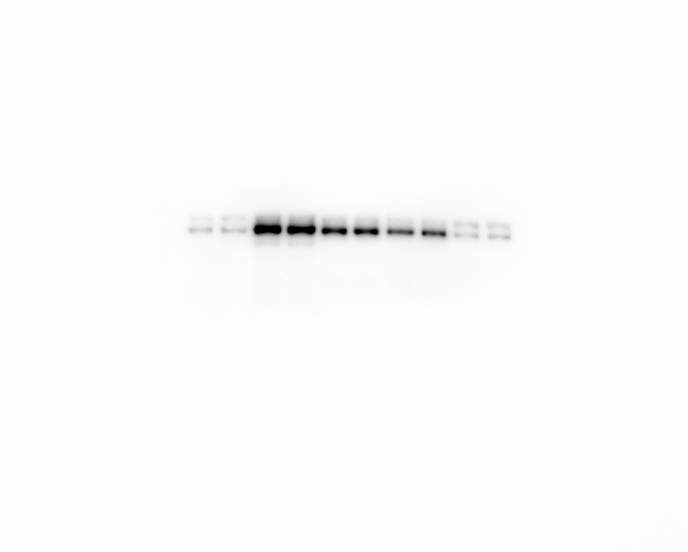


SREBP2


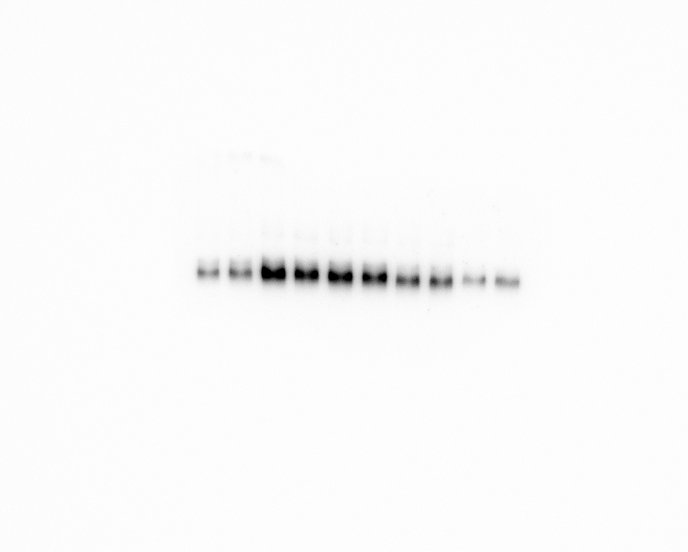


ACC


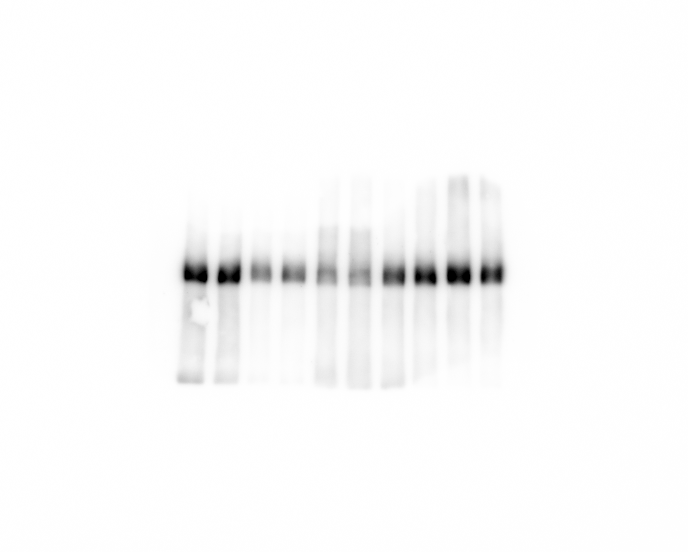


p-ACC


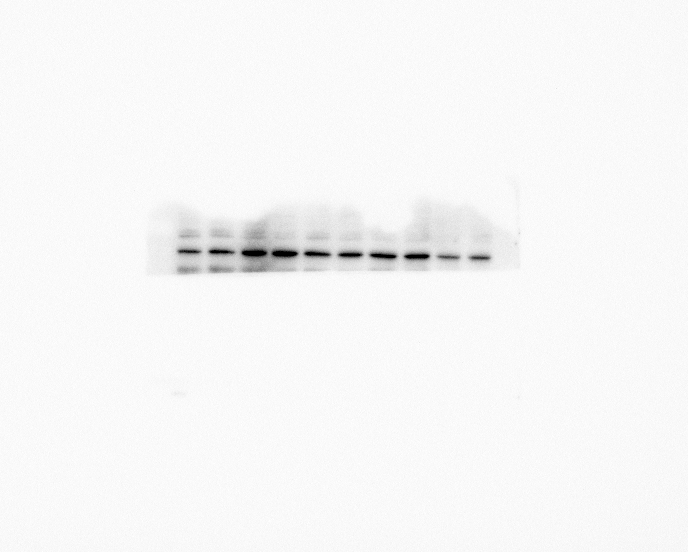


GSK3β


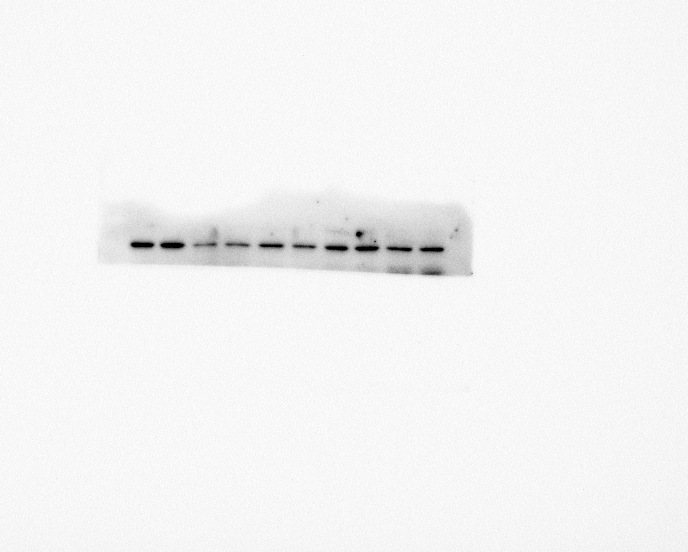


p-GSK3β

Supplement: S7 File — (DOC) [file pone.0195609.s008.doc]
