# Supplementary material for: Combined intervention of swimming plus metformin ameliorates the insulin resistance and impaired lipid metabolism in murine gestational diabetes mellitus
Source: PLoS One. 2018 Apr 20;13(4):e0195609. doi: 10.1371/journal.pone.0195609 (PMC5909919; doi:10.1371/journal.pone.0195609)
Supplement: S5 File — (DOC) [file pone.0195609.s006.doc]

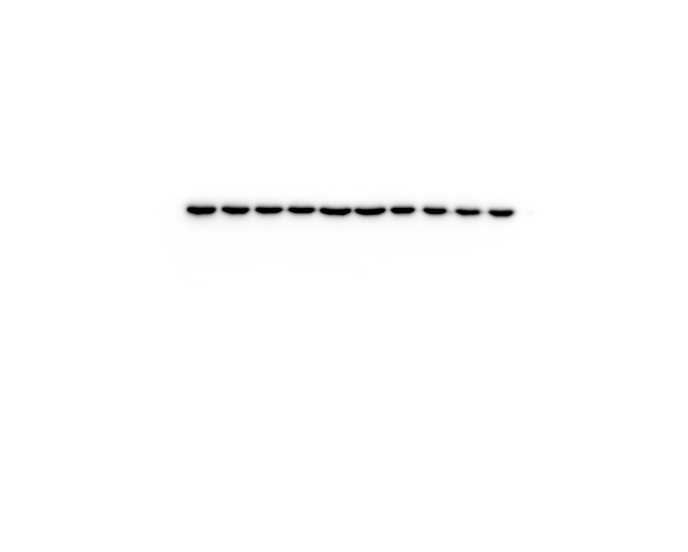


**β-actin for PEPCK**


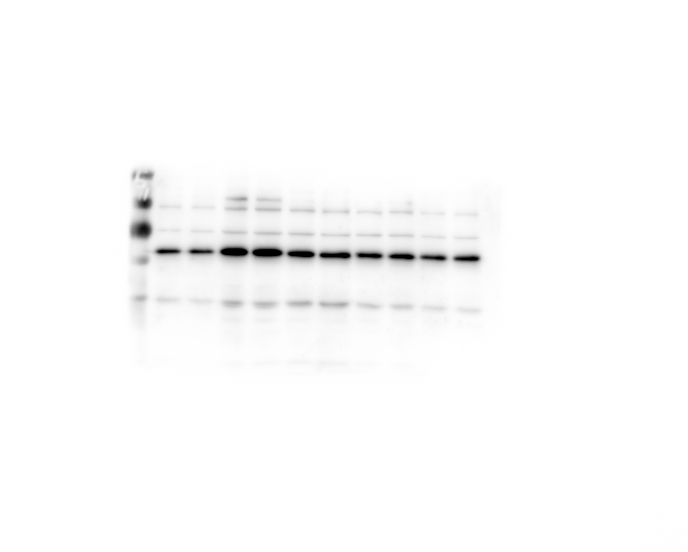


**PEPCK**


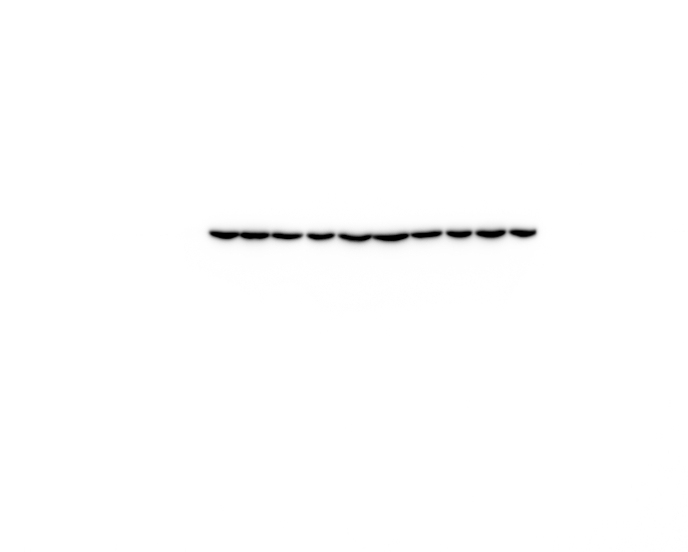


**β-actin for G6Pase**


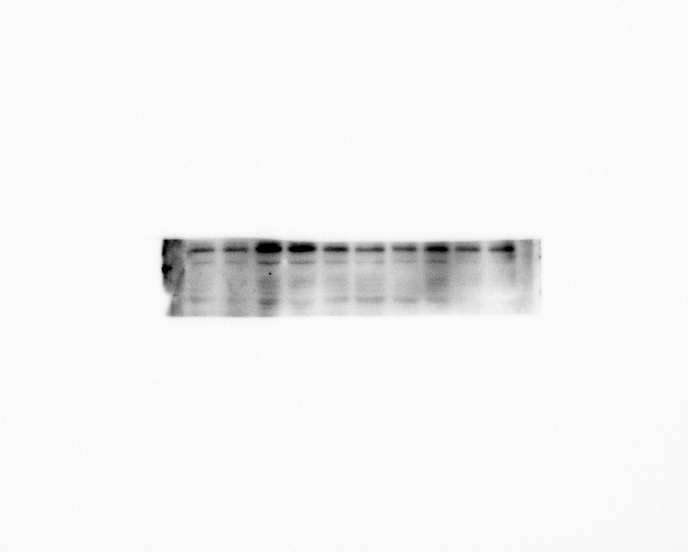


**G6Pase**

Hepatic β-actin mRNA Ct value

| **NC** | **DC** | **DS** | **DM** | **DSM** |
| --- | --- | --- | --- | --- |
| 16.52 | 16.57 | 17.11 | 16.99 | 16.59 |
| 16.73 | 16.67 | 16.93 | 16.98 | 16.42 |
| 16.53 | 16.74 | 16.86 | 16.89 | 16.56 |

Hepatic PEPCK mRNA Ct value

| **NC** | **DC** | **DS** | **DM** | **DSM** |
| --- | --- | --- | --- | --- |
| 16.96 | 15.17 | 15.31 | 16.15 | 17.31 |
| 16.97 | 14.69 | 15.54 | 16.56 | 17.26 |
| 16.85 | 14.97 | 15.41 | 16.24 | 17.48 |

Hepatic G6Pase mRNA Ct value

| **NC** | **DC** | **DS** | **DM** | **DSM** |
| --- | --- | --- | --- | --- |
| 19.42 | 16.14 | 17.17 | 17.83 | 19.10 |
| 20.27 | 16.38 | 16.95 | 18.00 | 19.12 |
| 19.25 | 16.35 | 16.79 | 17.99 | 19.03 |
